# Impact of thyroid autoantibodies on pregnancy: A prospective cohort study

**DOI:** 10.6026/973206300214749

**Published:** 2025-12-15

**Authors:** Yashika Sheetal, Mallikarjun Samala, Rahul Tiwari, Heena Dixit, Anil Managutti, Deepak Sharma, Akriti Mahajan

**Affiliations:** 1 Department of Obstetrics and Gynaecology, Saraswati Medical College, Atal Bihari Vajpayee Medical University, Unnao, Lucknow, Uttar Pradesh, India; 2 Piedmont Rockdale Hospital & Family, First Primary Clinic, Atlanta, GA, USA; 3 Department of Oral and Maxillofacial Surgery, Narsinhbhai Patel Dental College and Hospital, Sankalchand Patel University, Visnagar, Gujarat, India; 4 Department of Medical Health Administration, Index Institute, Malwanchal University, Index City, Nemawar Road, Indore, Madhya Pradesh, India; 5 Department of Oral medicine and radiology, Desh Bhagat Dental College and Hospital, Mandi Gobindgarh, Punjab, India

**Keywords:** Thyroid autoantibodies, pregnancy complications, pregnancy-induced hypertension, gestational diabetes mellitus, neonatal intensive care units

## Abstract

We conducted a prospective cohort study of 1,200 pregnant women recruited in early first trimester to evaluate the impact of thyroid autoantibodies (TPOAb and/or TGAb) on maternal and neonatal outcomes. Overall, 18.2% were antibody-positive. Compared with antibody-negative women, those with thyroid autoimmunity had significantly higher risks of pregnancy-induced hypertension (12.6% vs 7.9%), gestational diabetes (15.3% vs 10.2%) and neonatal intensive care unit admission (11.2% vs 6.5%). Adjusted logistic regression confirmed independent associations after controlling for maternal age, BMI, parity and TSH levels. Neonates born to antibody-positive mothers had lower mean birth weight (2980 ± 420 g vs 3125 ± 395 g, p=0.02) and higher risk of small-for-gestational age (8.7% vs 4.9%). Thus, we show that thyroid autoantibodies, even in euthyroid women, are associated with adverse pregnancy outcomes and justify closer monitoring during antenatal care.

## Background:

Thyroid autoimmunity (TAI), characterized by circulating thyroid peroxidase antibodies (TPOAb) and/or thyroglobulin antibodies (TGAb), is the most common autoimmune condition affecting women of reproductive age. Its prevalence in pregnancy ranges from 10% to 20% depending on geography and iodine status [1, 2]. Even in euthyroid women, thyroid autoantibodies may contribute to adverse pregnancy outcomes via mechanisms including placental inflammation, endothelial dysfunction and inadequate thyroidal adaptation to gestational demands [3, 4- 5]. Prior meta-analyses and large prospective cohorts have linked TAI to miscarriage, pregnancy-induced hypertension (PIH), preeclampsia, gestational diabetes mellitus (GDM), preterm birth and neonatal intensive care unit (NICU) admissions [6, 7, 8- 9]. However, results remain heterogeneous across populations. Some cohorts demonstrate modest but significant associations [10], while others report no meaningful differences once thyroid function is controlled [11]. The conflicting evidence leaves uncertainty about whether thyroid autoimmunity itself, independent of thyroid dysfunction, adversely impacts pregnancy. Moreover, data from South Asian populations remain limited and most evidence comes from East Asian or European cohorts [12]. Given possible ethnic and environmental modifiers of autoimmunity, region-specific evidence is critical. Therefore, it is of interest to describe the impact of thyroid autoantibodies on maternal and neonatal outcomes in a prospective cohort of pregnant women, with careful adjustment for potential confounders.

## Materials and Methods:

## Study design and participants:

We conducted a hospital-based prospective cohort study between January 2022 and December 2024 at a tertiary care teaching hospital. Pregnant women aged 18-40 years attending their first antenatal visit (≤12 week's gestation) were invited. Exclusion criteria: multiple gestations, pre-existing overt thyroid disease, diabetes mellitus, hypertension, or autoimmune disorders other than TAI.

## Data collection:

Baseline demographic and clinical data were collected. Blood samples were drawn for TSH, free T4, TPOAb and TGAb using chemiluminescent immunoassays. Women were categorized as TAI-positive if TPOAb and/or TGAb exceeded reference thresholds.

## Outcomes:

Participants were followed until delivery. Maternal outcomes included PIH, preeclampsia/eclampsia and GDM (diagnosed by IADPSG criteria). Neonatal outcomes included preterm birth, birth weight, small for gestational age (SGA), macrosomia, Apgar <7 at 5 min and NICU admission.

## Sample size and analysis:

A sample of 1,200 women provided 80% power to detect an odds ratio of 1.5 for PIH with α=0.05. Logistic regression was used for associations between TAI and outcomes, adjusted for maternal age, BMI, parity and TSH. A p<0.05 was considered significant.

## Results:

Of the 1,200 women enrolled, 218 (18.2%) were antibody-positive. Baseline age (mean 28.4 vs 27.9 years, p=0.3) and BMI (24.5 vs 24.2 kg/m^2^, p=0.4) were comparable between groups. In our cohort, thyroid autoantibody-positive women experienced a higher frequency of maternal complications compared with antibody-negative women. Pregnancy-induced hypertension was significantly more common (12.6% vs 7.9%) and gestational diabetes occurred in 15.3% compared with 10.2% of controls. Logistic regression confirmed that TAI was independently associated with PIH (OR 1.62, 95% CI 1.05-2.48) and GDM (OR 1.59, 95% CI 1.04-2.44), even after adjusting for age, BMI and baseline TSH levels (Table 1 - see PDF). Neonatal outcomes were also less favorable in the TAI-positive group. Birth weight was significantly lower (2980 ± 420 g vs 3125 ± 395 g), with higher rates of small-for-gestational age (8.7% vs 4.9%). The risk of NICU admission was almost doubled (11.2% vs 6.5%), reaching statistical significance (OR 1.82, 95% CI 1.07-3.11). Although preterm birth rates were higher among antibody-positive mothers (9.7% vs 7.3%), the difference did not achieve statistical significance (Table 2 - see PDF).

## Discussion:

This prospective cohort study demonstrates that pregnant women with thyroid autoantibodies have higher risks of PIH, GDM, NICU admission and lower neonatal birth weight compared with antibody-negative women, independent of thyroid hormone levels. Our findings align with recent large cohorts from China and Europe [1, 6 and 8], which reported modest but significant elevations in adverse outcomes among TAI-positive women. The adjusted odds ratios for PIH (~1.6) and GDM (~1.5) in our cohort are within reported ranges [7, 9]. Although preeclampsia risk was higher in antibody-positive women, this did not reach significance, likely due to sample size limitations. Larger studies have shown a clearer link between TPOAb positivity and preeclampsia [2, 10]. Our findings nonetheless suggest a trend toward increased severe hypertensive disorders. The biological plausibility is supported by mechanisms such as subclinical placental inflammation and impaired angiogenesis triggered by autoimmune activity [4, 12]. Additionally, TAI may limit thyroidal reserve, resulting in subtle hypothyroxinemia under pregnancy stress despite normal baseline thyroid function. This could contribute to impaired glucose metabolism, hypertension and fetal growth restriction. Strengths of this study include prospective design, standardized assays and adjustment for key confounders. Limitations include single-center recruitment, lack of long-term child follow-up and modest sample size for rare outcomes such as eclampsia or stillbirth. From a clinical perspective, these findings underscore the importance of screening for thyroid autoantibodies in early pregnancy. While current guidelines recommend against universal screening, our results suggest that identifying TAI-positive women may allow for intensified monitoring, lifestyle interventions and targeted research into levothyroxine or selenium supplementation strategies [13- 14]. Future research should address whether therapeutic interventions in euthyroid antibody-positive women can reduce adverse outcomes and whether antibody titers, rather than binary positivity, provide better risk stratification.

## Conclusion:

Thyroid autoantibody positivity is associated with increased risks of PIH, GDM, NICU admission and reduced birth weight. Early pregnancy screening and vigilant monitoring of antibody-positive women may improve maternal and neonatal outcomes. However, further randomized trials are needed to establish effective interventions.
